# Tracking novel visual word learning via different methods with an original FPVS-EEG approach

**DOI:** 10.3389/fnhum.2025.1647925

**Published:** 2025-10-20

**Authors:** Amaury Barillon, Christine Schiltz, Aliette Lochy

**Affiliations:** Department of Behavioral and Cognitive Sciences, Faculty of Humanities, Social and Educational Sciences, Institute of Cognitive Science and Assessment, University of Luxembourg, Esch-sur-Alzette, Luxembourg

**Keywords:** EEG-FPVS, word representation, novel words learning, reading, semantic

## Abstract

Reading is a crucial human skill and learning novel written word forms is a life-long process. Here, we tracked the emergence of novel word lexical and neural representations after a training procedure, contrasting two learning methods, in 32 monolingual adults. Half of the novel words were provided with orthographic and phonological information (OP), and half with additional explicit semantic information (OPS). At the neural level, we demonstrate for the first time the sensitivity of EEG recordings with fast periodic visual stimulation (FPVS) to track novel visual word learning. We used an oddball paradigm, with base stimuli (pseudowords) displayed at 10 Hz with deviant stimuli (words) every fifth item (at 2 Hz), in which word-selective responses at 2 Hz demonstrate lexical discrimination. While at pre-test, novel words were not discriminated, results show clear word-selective responses over the left occipital-temporal cortex (VOTC) post-learning with both methods. This finding suggests the creation of orthographic representations for novel words and fits with current views that this region is specialized for the rapid recognition and fast learning of novel word forms. Moreover, the behavioral lexical decision data reveal significant increases in reaction times after learning, for novel words’ lexical neighbors, which suggests lexical engagement through competition arising from newly formed representation. Contrary to our expectations, no advantage was found for the OPS method. Instead, results show stronger behavioral and neural changes with the OP method. In the discussion, we highlight possible reasons for this unexpected finding. First, the current implementation of the OPS method displaying simultaneous images and words during learning could have dragged the participant’s attention away from the orthographic form. Second, the speed of presentation of stimuli might have been too fast to allow fast semantic retrieval. Finally, semantic learning might have a different timeframe than word form learning, and the current findings would reflect only the latter. Our results nevertheless highlight the rapid emergence of new word-form representations, captured by the EEG-FPVS approach.

## 1 Introduction

Humans have the remarkable ability to learn novel words during their whole life, to memorize them, and to retrieve them from a mental dictionary or lexicon. However, unlike a dictionary, the mental lexicon is constantly updating connections between words and comprises several forms of representations: how the word is pronounced (phonological representation), how it is written (orthographical representation), and what it means (semantic representation).

When reading, once a novel word form has been encountered and decoded several times, it creates memory traces (Share, 1995) of its written form considered by some theoretical proposals as representations within an orthographic lexicon, which may but does not obligatorily activate semantic information (Coltheart et al., 2001; but see Harm and Seidenberg, 2004). Indeed, neuropsychological data have shown some case-studies of patients who display normal access to the lexicon (recognizing the orthographic word-form, being able to read it out loud correctly), without having access to its meaning or semantics (e.g., Raymer and Berndt, 1996; Purcell et al., 2014). However, in non-clinical settings, whether simple repeated visual experience relating orthography to phonology is sufficient to create the creation of novel representation and the integration of new words in the lexicon, is still a matter of debate.

Some perspectives consider indeed that one should distinguish two distinct processes during the formation of new lexical traces: lexical configuration and lexical engagement (Leach and Samuel, 2007). Lexical configuration can be conceived as the stage when becoming familiar with a word by gathering global information on its form, including its orthographical and phonological representation, whereas lexical engagement refers to the integration of the novel word in the lexicon, reflected by the interaction (competing or facilitating effects) with previously known words. Only lexical engagement induces competition effects between the novel word (e.g., BANARA), and pre-existing neighbor words (e.g., BANANA), which manifests as a delay in response times when judging those pre-existing neighbors (Bowers et al., 2005; Gaskell and Dumay, 2003; Qiao et al., 2009; Qiao and Forster, 2013). The novel (trained) words are thought to compete with the pre-existing words for activation during a lexical decision task, which results in slower reaction times due to the inhibition of the novel lexical representation (Grainger and Jacobs, 1996; McClelland and Rumelhart, 1981). Thus, lexical engagement is considered evidence for the integration of novel words into the orthographic lexicon. Importantly, novel word recognition alone may reflect episodic memory rather than lexicalization. Supporting this distinction, studies in both spoken (Davis and Gaskell, 2009; Dumay and Gaskell, 2007; Gaskell and Dumay, 2003; Henderson et al., 2014) and visual (Bakker et al., 2015) word recognition demonstrate that immediate learning is not sufficient for lexical engagement, which typically requires a period of consolidation. Actually, when tested immediately after learning, novel words like *cathedruke* (a phonological neighbor of *cathedral*) were even shown to produce a facilitatory effect on familiar neighbors (−27 ms, Gaskell and Dumay, 2003), while the effect reversed after consolidation inducing slower decision times (Dumay and Gaskell, 2007). The initial facilitatory effect is speculated to stem from activation of the representation of the closest real words (neighbors) during learning, while inhibitory competition emerged as the novel word became more robustly engaged at the lexical level. In other words, lexical configuration may occur immediately, whereas lexical engagement requires consolidation. However, other studies reported that consolidation was not necessary to observe the effects of lexical engagement (Fernandes et al., 2009; Geukes et al., 2015; Kapnoula et al., 2015; Kapnoula and McMurray, 2016; Lindsay and Gaskell, 2013; Szmalec et al., 2012; Walker et al., 2019).

Besides the role of consolidation, the quality of learning and particularly the availability of semantic representations, has been extensively studied. Learning a word associated with a clear meaning is expected to strengthen its integration into the lexicon. Indeed, a large body of research suggests that adding semantic form further improves learning of novel words (Ehri, 2013; Mimeau and Ricketts, 2018; Ouellette and Fraser, 2009; Seidenberg and McClelland, 1989). The triangle model of word recognition (Seidenberg and McClelland, 1989) considers that phonology, orthography, and semantics are typically activated when reading a word, thereby aiding the process of word recognition. The lexical quality hypothesis (Perfetti and Hart, 2008) also emphasizes that the quality of these representations is crucial to rapidly and reliably acquire new lexical entries. In that perspective, readers depend on the diversity of forms to integrate new lexical items into the lexicon. Given that a high-quality representation is constituted by tightly connected orthographic, phonological and semantic information, the knowledge of one type of information should facilitate the learning of another type of information, and hence of the novel word representation (Perfetti, 2007). Thus, the combination of orthographic and semantic training typically results in better lexicalization of novel words (Ricketts et al., 2011; Rosenthal and Ehri, 2008). However, while semantic content is often included in training protocols or assessment tasks, current evidence suggests that semantic information alone does not drive lexical competition. For instance, Bowers et al. (2005) emphasized that learning a novel word form like *BANARA* provides no semantic cues for classifying *BANANA* and argued that the observed competition must arise from form-based interference. This interpretation is supported by Chen and Mirman (2012), who proposed that lexical competition is driven primarily by orthographic and phonological overlap, not by shared meaning.

Behavioral responses provide valuable insights on visual word recognition, but they remain indirect measures and subject to interpretation. Slower reaction times may for instance reflect competition effects within the lexicon or rather result from decisional processes and hesitation. In contrast, neuroimaging techniques can track brain activity online during word recognition. It is well established that the left hemisphere and more specifically the left ventral occipito-temporal cortex (VOTC) plays a crucial role in visual word recognition (Dehaene et al., 2002; Lochy et al., 2018; Price and Devlin, 2011; Seghier et al., 2012). Originally identified by Cohen et al. (2000) as a region consistently activated during reading, the Visual Word Form Area (VWFA) has since been implicated in a range of processes, including word recognition (McCandliss et al., 2003; Pugh et al., 2001), reading acquisition (Dehaene et al., 2010; Dehaene-Lambertz et al., 2018), and novel word learning (Glezer et al., 2015; Riesenhuber and Glezer, 2017; Tao et al., 2024). Although controversial, this region has been described by some authors as a “visual dictionary,” functioning as an orthographic lexicon that contains highly selective neuronal representations for individual real written words (Glezer et al., 2009, 2019), distinct from the orthography-to-semantic interface (the Basal Temporal Area, BTLA) or more anterior regions dedicated to semantic processing (Anterior Temporal Pole, ATL) (see Purcell et al., 2014). According to this framework, learning new words should lead to a selective increase in neural specificity within the VWFA (Glezer et al., 2015; Riesenhuber and Glezer, 2017). Indeed, studies have shown that the VWFA exhibits remarkable plasticity, with changes in neural tuning emerging rapidly, even after a small number of exposures and without explicit memorization instructions. For instance, word-like selectivity in the VWFA has been observed after only 5–6 exposures within a single scanning session, suggesting that novel words can be integrated into the brain’s visual lexicon with surprising efficiency (Glezer et al., 2015). Before training, pseudowords typically elicit tuned responses in the VWFA. However, following exposure, trained pseudowords evoke tightly tuned responses comparable to those for familiar real words, while untrained pseudowords continue to elicit diffuse activation. These findings underscore the VWFA’s critical role in orthographic learning and its ability to rapidly adapt to support the representation of newly learned words.

Other neuroimaging techniques such as Event-Related Potential (ERP) studies have also addressed neural changes associated with novel word learning. The Late Positive Component (LPC), peaking around 600 ms, is associated with successful encoding of word form and meaning (Perfetti et al., 2005). The N400, linked to automatic semantic processing, is typically reduced for semantically congruent or learned items, reflecting facilitated meaning access (Bentin, 1987; Kiefer and Brendel, 2006; Kutas and Hillyard, 1984; Perfetti et al., 2005; Van Petten, 1993). Also, the P200 component, involved in orthographic processing, has been shown to be modulated for novel word forms without meanings, implying the formation of an orthographic representation (Bermúdez-Margaretto et al., 2020). However, like in fMRI or behavioral studies reviewed above, the debate remains open regarding the mandatory role of consolidation or semantic. First, the formation of lexical traces for novel words has been shown in some studies to be immediate (Bermúdez-Margaretto et al., 2020; Borovsky et al., 2010, 2012; Partanen et al., 2018), while others suggest the necessity of a period of consolidation (Bakker et al., 2015; Davis and Gaskell, 2009). Second, some authors suggest that new neural traces for novel words can emerge through sole orthographic learning (Bermúdez-Margaretto et al., 2015, 2020; Partanen et al., 2018) while others imply mandatory semantic learning (Angwin et al., 2014; Bermúdez-Margaretto et al., 2018; Borovsky et al., 2012; Frishkoff et al., 2010; Perfetti et al., 2005).

Despite extensive research, results thus remain inconclusive: findings vary across studies, the timing of lexical integration remains debated, and the contribution of semantic enrichment is still uncertain. To address these questions, we applied frequency-tagging (Fast Periodic Visual Stimulation, FPVS) with EEG recordings. This approach has the advantage of measuring and quantifying neural word-selective responses that are automatic and unintentional, as it does not require any explicit linguistic task. Indeed, the paradigm involves presenting periodic visual stimuli, leading to periodic neural responses known as steady-state visual evoked potentials (SSVEP), a concept initially introduced by Regan (1966). Since then, it has been adapted to measure visual discrimination of faces (Liu-Shuang et al., 2014; Rossion and Boremanse, 2011) and visual word recognition (Lochy et al., 2015, 2018; Lochy et al., 2024; Volfart et al., 2021) because of its high sensitivity (i.e., high Signal-to-Noise Ratio, SNR) (Norcia et al., 2015; Regan, 1989) and objectivity (i.e., frequencies of interest are determined *a priori*). In the so-called oddball paradigm, base stimuli (e.g., pseudowords) are presented at a fast rate (for instance, 10 Hz), and deviant or oddball stimuli (e.g., words) are periodically inserted in the stream (for instance, every 5 items, thus at 10 Hz/5, 2 Hz). A neural response at the oddball frequency (2 Hz and harmonics) reflects the brain’s ability to implicitly discriminate words from pseudowords, without any explicit linguistic task. For instance, Lochy et al. (2015) found robust discrimination responses for written words within pseudofonts, nonwords, or pseudowords over the left occipito-temporal cortex. Interestingly, the amplitude of the word-selective response was modulated by the wordlikeness of base stimuli, being stronger for a coarse contrast (words in pseudofonts or nonwords) than for the finer pseudowords/words contrast. The recorded response thus reflects a differential processing between the two stimulus categories and corresponds to the specific processes triggered for the oddball category, over and above the common processes for the two stimulus categories. In this regard, responses for words within pseudowords are interpreted as reflecting lexical processing (Lochy et al., 2018, 2024; Marchive et al., 2025; Hauk et al., 2025). Recently, this approach has also proven its sensitivity to probe semantic categorization of visual words (Volfart et al., 2021). In that study, words of one semantic category were used as base stimuli (e.g., animal names), and words of another category as deviant stimuli (e.g., cities names). Sensitivity to semantic features provides important information concerning the validity of the FPVS-EEG as a tool for measuring all levels of word learning and especially the role of the semantic dimension when learning novel words.

**Figure 1:**
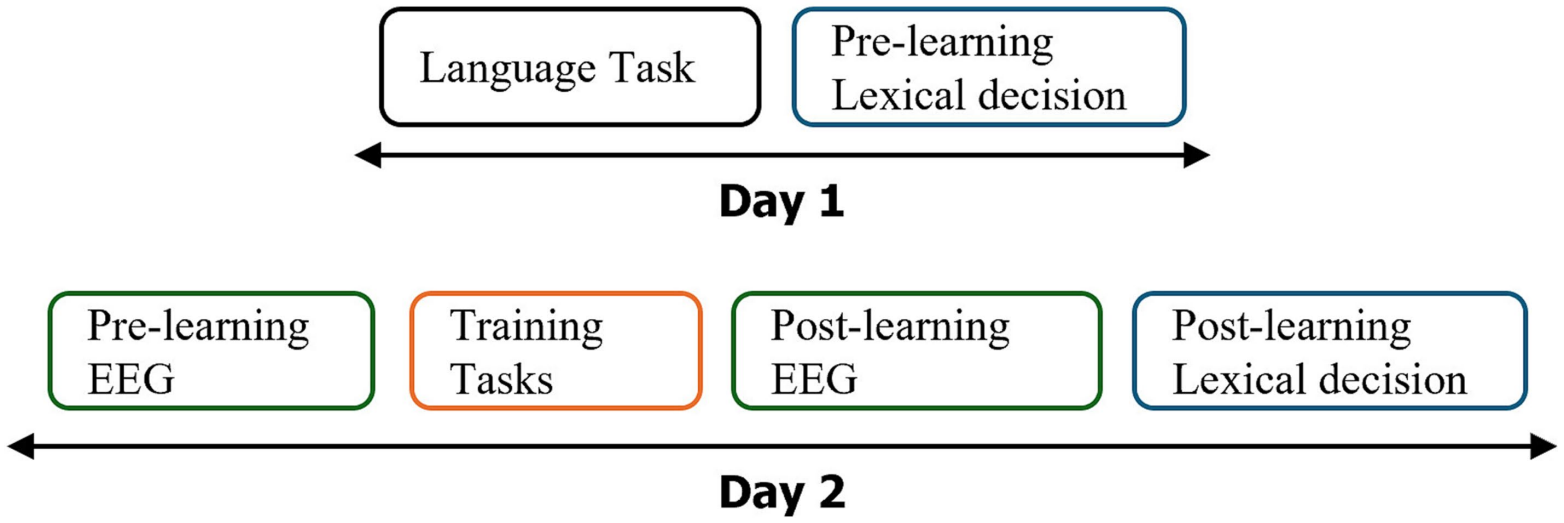
Design of the experiment: the experiment was conducted over 2 days using a test–retest design. Tasks are color-coded: Black for language tasks, blue for the Lexical Decision Task (LDT), green for EEG-FPVS recordings, and orange for the learning phase involving the 32 novel words. See Figure 2 for a detailed breakdown of the training tasks.

In the present study, participants learned 32 novel words: half with full semantic representations and half with only orthographic/phonological form. To assess lexicalization, that we view here as the creation of novel word representations in the orthographic lexicon, we used a test–retest design combining behavioral and neurophysiological measures (see Figure 1). Lexical engagement was evaluated through a lexical decision task (LDT; Rubenstein et al., 1970), administered before and after learning to examine the influence of newly learned words on their orthographic neighbors. We also included pseudoword neighbors differing by one letter to test for potential interactions. Neural evidence for the creation of novel word representations was assessed with Fast Periodic Visual Stimulation EEG (FPVS-EEG), using an oddball paradigm in which novel words were inserted among pseudowords at a rate of 2 Hz. Our first objective was to determine whether novel visual word learning can be observed immediately after short training tasks, without consolidation. Behaviorally, we expected first, improved word recognition (higher accuracy). Second, if the novel representations started to be integrated in the lexicon and interact with previous knowledge, then we expected a competing effect on orthographic neighbors, classical marker of lexical engagement (e.g., slower responses to *BANANA* after learning *BANARA*). As concerns neurophysiological measures, we expected no word-selective responses for novel words at pre-test (they would not be distinguished from pseudowords), but the emergence of discrimination responses at post-test over the left occipito-temporal cortex (Lochy et al., 2015) indicating the creation of novel word representations. To our knowledge, this study is the first to use a FPVS paradigm to investigate novel word acquisition. Concordant findings across behavioral and neural measures would support the rapid integration of novel words rather than episodic traces of words.

Our second objective was to examine the impact of providing meaning, or not, on the acquisition of novel visual words. Specifically, we contrasted two types of learning methods: one involving the presentation of all constituent representations (orthographic, phonologic, and semantic) of novel words (OPS), and the other one providing limited information by presenting only the orthographic and phonologic (OP) representations. We expected that the OPS method would lead to better lexical integration, reflected behaviorally by a stronger competing effect on neighbor words in the lexical decision task, and at the neural level by greater EEG amplitudes for the word-selective responses compared to words learnt with the OP method.

## 2 Materials and methods

### 2.1 Participants

Our sample size of 32 participants, tested at [MASKED], provided sufficient power for our analyses and is consistent with previous EEG studies in this domain (e.g., Lochy et al., 2015, 2024). All participants were right-handed adults (21 females; mean age = 21.78 years, age range = 18–32 years). They were French native or did their education in French and had normal/corrected to normal vision. They were informed that they would be taught new existing, but very rare words in the recruitment procedure. All participants signed a consent form prior to the study and financial compensation was given for their participation. This research was approved by the Ethical Committee of the University of [MASKED] and conforms to the Declaration of Helsinki.

### 2.2 General design

The experiment was designed over 2 days at an interval of 1 week (see Figure 1). During the first day, general cognitive and language abilities were assessed with different behavioral tests. A lexical decision task was performed by participants to record their pre-test reaction times and to ensure that they did not know our stimuli [rare French words: words to learn (novel words hereafter) and control words remaining unlearnt (unlearned words hereafter)]. The second day included a first EEG recordings session pre-learning, a learning task (32 novel words), and an immediate post-learning EEG recordings. During training, half of the novel words (*N* = 16) were learnt with orthographic and phonological forms (OP) and the other half (*N* = 16) with orthographic, phonological, and semantic information (OPS). Then the lexical decision task was performed again at the end of the second session (post-learning).

### 2.3 Stimuli

#### 2.3.1 Novel words

Our main stimuli consisted of 32 novel words, which were presented in lexical decision tasks, training tasks, and EEG tasks (see Table 1 for examples, presented per task). We selected real but extremely rare French nouns that were unknown to participants. Since participants were required to learn these words, they were labelled as “Novel Words,” followed by OP or OPS to indicate the training method used (e.g., novel word OP, or novel word OPS). The stimuli were selected from the Lexique 3.83 database (New et al., 2004) and/or Wordgen (Duyck et al., 2004). The novel words list was divided into two matched subsets (A & B), with each word being learned under either OP or OPS training, counterbalanced across participants. This ensured that the same word was learned with OP training for half of the participants and OPS training for the other half. The two lists were controlled for lexical properties to ensure comparability, including number of letters (*p* = 1, min = 4, max = 6), number of syllables (*p* = 0.096, min = 1, max = 3), number of orthographic neighbors (*p* = 0.682, min = 1, max = 5), and bigram frequency (*p* = 0.843, min = 5,727, max = 17,361) (see Table 2).

**Table 1:** Example of stimuli used over the study: 4 items are given as examples, but 32 items were used in the study, divided into 2 sets (A & B).

Stimuli for Lexical decision task					
Novel words	Word orthographic neighbor (ON1L)	Pseudoword neighbor (PW1L)	Unlearned words	Filler words	Filler PW
*N* = 32	*N* = 32	*N* = 32	*N* = 32	*N* = 40	*N* = 40
*APION*	AVION	APIOR	APODE	EFFORT	GAMPIL
*FREUX*	CREUX	FREUS	LIPPE	DOIGT	LOCTON
*POTARD*	MOTARD	POTURD	VALINE	LUNCH	RIDAN
*ORONGE*	ORANGE	URONGE	TORON	EFFORT	PELART

Stimuli for the training tasks							
One set of 16 items are learnt with OPS and the other set with OP, counterbalanced across participants. Definitions are presented only in OPS training							
		Visual matching			Orthographic matching		
Novel words	Definition	PW1 (1L)	PW2 (1L)	PW3 (1L)	PW1	PW2	PW3
		*Differ by 1L from Novel word*			*Same pronunciation as Novel word*		
*APION*	A type of beetle (insect), including many weevils, and harmful to legumes.	ATION	UPION	ACION	HAPION	APPION	HAPPION
*FREUX*	A bird resembling a crow characterized by its narrow beak	VREUX	TREUX	FLEUX	FREUT	PHREU	FREU
*POTARD*	Pharmacist, pharmacy technician or student in pharmacy	POFARD	POSARD	FOTARD	POTAR	PAUTARD	POTART
*ORONGE*	Mushroom characterized by its beautiful yellow-orange cap	ORORGE	ORONSE	ORUNGE	ORONJE	HORONGE	AURONGE

*Stimuli for the EEG task*					
Novel words learnt with OP, novel words with OPS, known words and Unlearned words were presented in FPVS-EEG sequences as deviant among matched pseudowords					
		EEG	EEG	EEG	EEG
		PW1 (CV)	PW2 (CV)	PW3 (CV)	PW4 (CV)
		*Keep the consonant-vowel structure of matched words*			
Novel words	*APION*	AROUS	ALOIN	AMOIR	APIEL
	*FREUX*	TROUP	DREIT	CHIOL	FREIL
	*POTARD*	CUSARD	VETARD	TOMARD	RUTARD
	*ORONGE*	ADELTE	ATONDE	URASSE	AVANDE
Known words	VILLA	NADRE	TIVRE	BERDE	ROSTE
	CHIEN	TREUR	CROUS	TROUF	DRAIS
	CANAL	BULAN	BACAL	ZOLER	TOMEL
	CHOSE	CRASE	CHIRE	BRIGE	GLITE
Unlearned words	APODE	ADOSE	AGIPE	UMAGE	ARURE
	LIPPE	SELVE	VADRE	DOCLE	TUFLE
	VIRURE	LIPURE	MADURE	BADITE	MUTURE
	TORON	PENIN	DOTIN	LIBON	MANOR

**Table 2:** Characteristics for stimuli of set A and set B used in lexical decision task.

	SET A				SET B			
	Novel Words A	PW1L	ON1L	Unlearned	Novel Words B	PW1L	ON1L	Unlearned
*N*	16	16	16	16	16	16	16	16
N. Ortho Neighbors	2.75 (0.33)	2.56 (0.32)	5.625 (0.8)	1.375 (0.221)	2.57 (0.30)	2.375 (0.41)	3.125 (0.42)	2.375 (0.706)
Bigram frequency	11,915 (964)	11,875 (971)	14,191 (961)	12,452 (1452)	11,671 (855)	11,727 (870)	12,350 (916)	12,590 (1425)
Lexical frequency	0.39 (0.12)		55 (19)		0.40 (0.12)		64 (26)	

Characteristics for stimuli of set A and set B used in lexical decision task: novel words, 1-letter close pseudowords (PW1L), 1-letter close orthographic neighbors (ON1L) and Unlearned words. The table provides the mean and SD (in parenthesis) for the number of orthographic neighbors (N. Ortho neighbors), bigram frequency, and lexical frequency (per million, CELEX database).

#### 2.3.2 Pseudowords neighbors (PW1L)

Pseudoword neighbors are used in the LDT to assess the impact of learning novel words on similar letter-strings forms. To create the 32 pseudowords neighbors (PW1L), one letter was changed from each novel word (e.g., novel word: *APION*, PW1L: *APIOR*) while maintaining pronounceability and compliance with French orthographic rules. These pseudowords neighbors were matched listwise to novel words on bigram frequency (*p* = 0.79) and the number of orthographic neighbors (*p* = 0.51, see Table 2).

#### 2.3.3 Word orthographic neighbors (ON1L)

Real word orthographic neighbors are used in the LDT to assess the impact of learning novel words on lexical engagement (slower reaction times at post-test). Word orthographic neighbors consisted in existing French words which differed from the novel words by one letter [e.g., novel word: *APION*, ON1L: *AVION (plane)*]. They were selected based on their bigram frequency (*p* = 0.131; max = 21,183; min = 7,521, see Table 2), syllabic structure and lexical frequency (*p* = 0.81; max = 403; min = 0.88, see Table 2).

#### 2.3.4 Unlearned words

To have a set of neutral, baseline, stimuli, we used unlearned words both in the lexical decision task, and in the EEG task. They consisted of 32 very rare French words similar to novel words except they were not learned. They were matched to novel words in number of letters, number of orthographic neighbors (*p* = 0.65), and bigram frequency (*p* = 0.540).

#### 2.3.5 Known words

Known words served as a baseline control condition in the EEG task to establish word-selective responses for existing words. They were unrelated frequent French words and matched to the two novel words subsets in terms of syllabic structure, bigram frequency (Set A: *p* = 0.691; Set B: *p* = 0.849), consonant-vowel structure, and number of letters (Set A: *p* = 0.379; Set B: *p* = 0.603). These words were selected from the stimuli used in Lochy et al. (2024) and were frequent (*M* = 11,392; *SD* = 3,956) and regular.

#### 2.3.6 Pseudowords for EEG (CV structure)

In the EEG task, we presented words (novel words, unlearned words and known words) as deviant stimuli among pseudowords, to assess the discrimination response for words. For this contrast, we built lists of pseudowords on the basis each words’ syllabic structure on an item-by-item basis, maintaining the consonant-vowel structure and matching each list to the corresponding words’ list in terms of bigram frequency (*p* > 0.41) and number of orthographic neighbors (*p* > 0.35).

#### 2.3.7 Pseudowords for orthographic matching (same pronunciation)

For one of the training tasks, where participants had to choose the correct spelling (orthographic matching), pseudowords built to match the same pronunciation as novel words served (e.g., novel word*: APION,* PW same pronunciation*: HAPION, APPION, HAPPION*).

#### 2.3.8 Pseudowords for visual matching

One of the training tasks (visual matching) required participants to choose the word corresponding to target with a change in case (lower-case/upper-case), among 4 alternatives. Thus, we built 3 distractor pseudowords resembling the target novel words but differing by 1 letter.

### 2.4 Cognitive tests

To ensure that our participants did not present any language or cognitive disorders, we assessed them with the following standardized tests: words comprehension with the French Wechsler Adult Intelligence Scale-IV (WAIS IV) (subtest vocabulary); verbal episodic memory with the subtest Verbal Paired Associates of the MEM IV (French version of the Wechsler Memory test, 4th edition). The *Evaluation des Compétences en Lecture chez l’Adulte de plus de 16 ans* (ECLA 16+) (Gola-Asmussen et al., 2010) was used to assess reading ability with a meaningless text (Alouette) and with reading of pseudoword/regular/irregular word lists. Other subtests from the ECLA 16+ were: the digit span/backward digit span task (flexibility, working memory), spoonerism and phoneme suppression (phonological awareness and metaphonology).

## 3 Procedure

### 3.1 Lexical decision task

Participants were asked to determine whether the presented letter string was a word or a pseudoword, responding as quickly and accurately as possible. A short training block (4 items) was provided to familiarize them with the task and the response keys. Participants answered using “L” (word) and “S” (pseudoword), with counterbalanced key assignments across participants.

The training phase was followed by four blocks of 52 stimuli, for a total of 208 stimuli. Each block lasted approximately 160 s, with optional short breaks between blocks. Each trial began with a 500 ms fixation cross, followed by the stimulus, which remained on-screen until response. Afterward, a mask (#) appeared for 1,500 ms before the next trial. The entire task lasted approximately 13 min.

The stimuli included 32 novel words, 32 orthographic neighbors words (ON1L), 32 pseudowords neighbors (PW1L), and 32 unlearned words, as described previously. Additionally, 80 filler items (40 pseudowords and 40 words, half 5-letters, half 6-letters) were added to balance the lists. Word fillers were equally split between frequent/infrequent and regular/irregular. In addition, the word fillers served as a baseline for comparison (in Supplementary material) with orthographic neighbors (ON1L), while the pseudoword fillers were used as a baseline for comparison with pseudoword neighbors (PW1L).

All stimuli (words and pseudowords) were presented without diacritic marks, in bold black Arial font (size 24), on a gray background at the center of the screen.

### 3.2 Learning procedure

**Figure 2:**
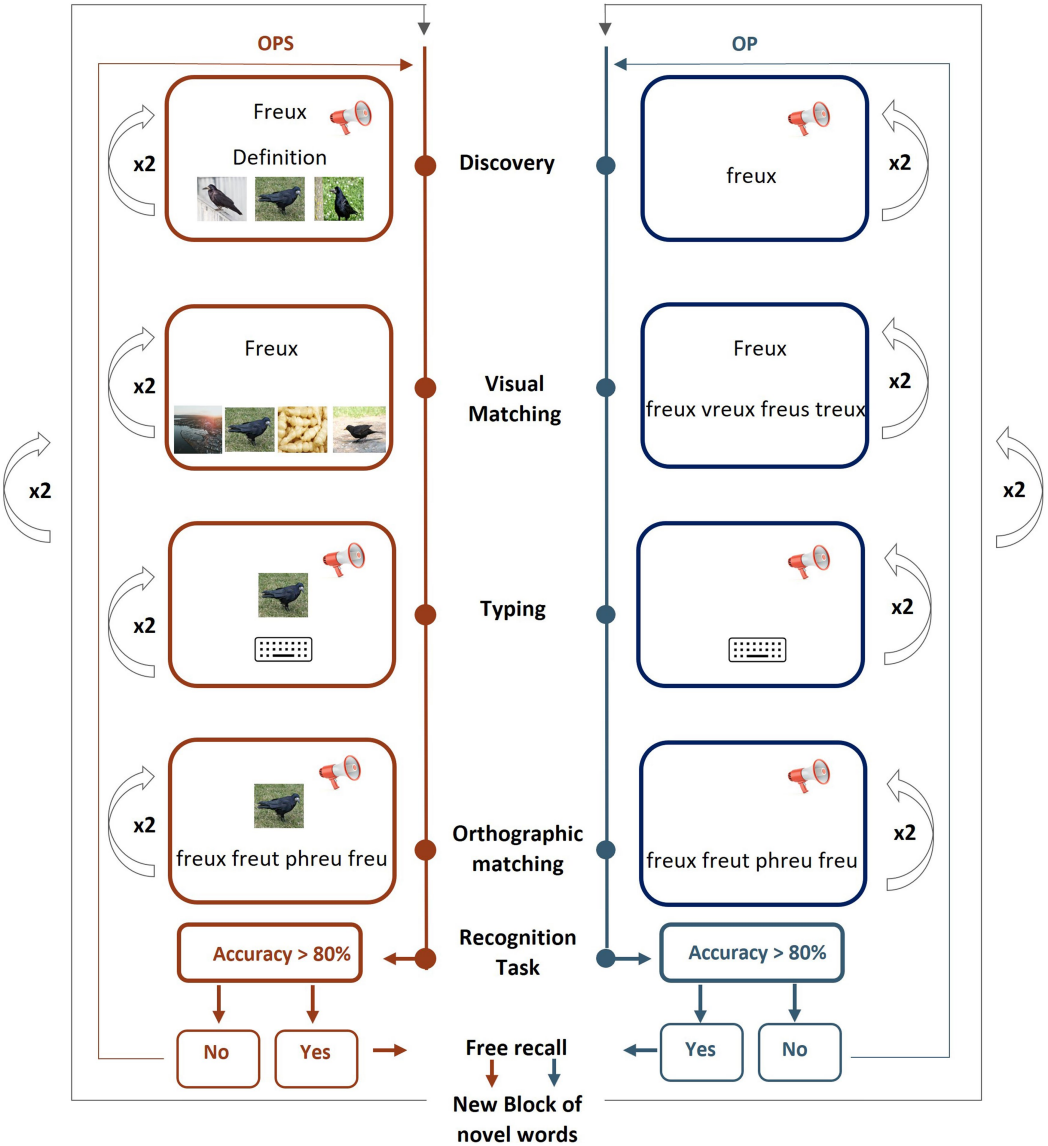
Training paradigm. Tasks are presented in blocked order with discovery as the first task and free recall, the last one. Four learning tasks are performed on 4 blocks of 8 items at a time with the OPS (Orthographic, Phonological and Semantic form) method (for 16 items, in red, left side) and the OP (Orthographic and Phonological form) method (for 16 items in blue, right side). Every task is repeated twice. Each written form is presented 6 times during training (no written presentation in the Typing task), 8 times in the feedback display, and once in the recognition task.

Each novel word subset was trained using one of two learning methods, randomly assigned and counterbalanced across participants. Training is visually depicted in Figure 2 and consisted of four learning tasks (*discovery, visual matching, typing, and orthographic matching*) and two evaluation tasks (*recognition task and free recall task*). All tasks (except for the free recall task, which was conducted on paper) were presented using E-Prime 2.0,^1^ with words displayed in lowercase, black bold Courier New font (size 18) on a grey background. During training, the two novel word subsets (*N* = 16 words) were further split into two blocks of 8 words to ease the learning, resulting in a total of four blocks. Within each learning task, words were presented in two loops of 8 stimuli in a pseudo-randomized order (no immediate repetition), ensuring that participants performed each task twice for a given set before proceeding to the next task. For all learning tasks, correct (green font) or incorrect (red font) feedback was provided after each response, regardless of the method. Both correct and incorrect feedback displayed the correct answer on the screen (e.g., *“Correct, the response was apion”* or *“Incorrect, the response was apion”*). In the semantic (OPS) training, an image of the corresponding object was also included in the feedback.

The four learning tasks (*discovery, visual matching, typing, and orthographic matching*) were presented in a blocked order and repeated twice for each block of 8 novel words (see Figure 2).

In total, each novel word was presented in its written form 6 times in training tasks, plus 8 times in feedback displays, and once in the recognition task for a total of 15 presentations.

#### 3.2.1 Discovery

The first learning task (discovery) involved the simultaneous presentation of the phonological and orthographic forms of each novel word in the OP method, while in the OPS method, a definition and corresponding image were also displayed on the screen (Figure 2). Novel words were randomized and displayed for 5 s before proceeding to the next trial.

To ensure consistency in auditory presentation, each of the 32 novel words was converted into audio format, with two versions (male and female voices) generated using the Acapela-Box online tool.^2^ The definitions were recorded using the same speech synthesis settings [*normal speech rate (0) and voice shaping (0)*]. The final auditory stimuli were saved in MP3 format **(**48 kbps, mono, 16-bit, 48 kbps), ensuring uniform acoustic quality across all recordings. The definitions of the novel words were adapted from various online dictionaries (Larousse, L’Internaute) to summarize their semantic features in a single sentence. The corresponding images were selected from free-license websites (Google Images, Pixabay, Pexels) and formatted to 240 × 240 pixels using Bulk Image Crop.

#### 3.2.2 Visual matching

The second task was a visual matching task using a 4-alternative forced choice (4AFC) format. The target novel word was presented in the top row (*y* = 10%), centered (*x* = 50%), and written in lowercase letters. The alternative to choose from were displayed at the bottom of the screen (*y* = 67%) in a horizontal line. Participants responded using the keyboard keys (C, V, B, N, AZERTY keyboard) with no time limit. The position of the correct answer varied from trial to trial. In the OP method, the bottom stimuli consisted of four-letters strings in capital letters. One was the target, and the others were 1-letter-close pseudowords (see Table 1 in General Stimuli). In the OPS method, the bottom stimuli consisted of four images. One corresponded to the target, one was a semantic distractor, two corresponded of another novel word.

#### 3.2.3 Typing

During the typing task, the phonological form of the novel word was presented auditorily, and participants were instructed to correctly write its orthographic form, with no time limit. The task was identical across methods, except that in the OPS method, the picture associated with the word was displayed on the screen under the written word.

#### 3.2.4 Orthographic matching

The fourth task consisted of an orthographic matching. Participants heard the phonological form of the novel word and were presented a new 4AFC task in which they had to choose the correct spelling of the Novel word. The four alternatives consisted in the correct target and 3 pseudowords that corresponded to plausible spellings of the novel word (see Table 1 in General Stimuli). The position of the bottom stimuli to choose from was similar to the visual matching task. Responses were made using the same keys as in the visual matching task, and there was no time limit for answering. The task was identical across both methods, except that in the OPS method, a picture depicting the object was also displayed.

#### 3.2.5 Recognition and free recall tasks

At the end of each block of four learning tasks, participants completed a recognition task without feedback, with a mandatory accuracy threshold of 80% to proceed. If the threshold was not reached, participants were required to repeat the four learning tasks for that block; however, all participants successfully met the criterion on their first attempt. During the recognition task, stimuli appeared in the center of the screen. Participants were instructed to press ‘S’ if they recognized the word and ‘L’ if they did not. Each novel word was presented along with its corresponding 1-letter pseudowords and pseudowords with same pronunciation, previously seen in the matching tasks. Thus, the task contained the eight novel words and their orthographic variations.

The final assessment, free recall, was conducted on paper. Participants were asked to recall and write down the eight novel words they had just learned. The task ended either after 1 min or once all words had been correctly recalled. The material used for the free recall was only a sheet of paper and a chronometer.

### 3.3 EEG task

**Figure 3:**
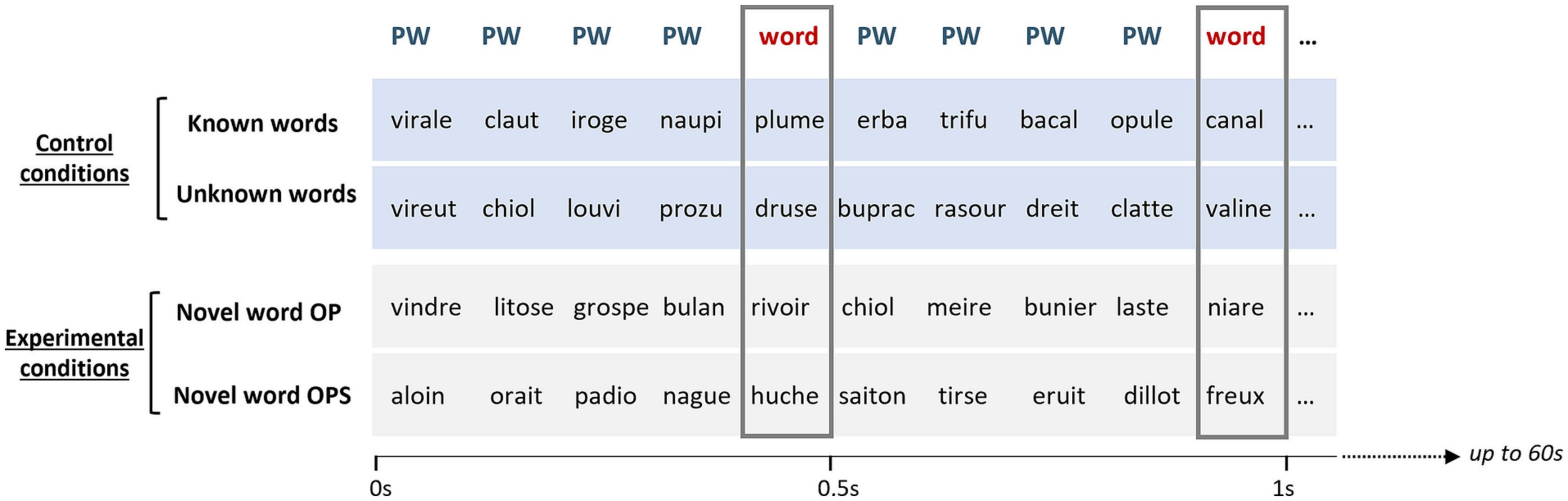
Illustration of the four conditions tested in FPVS. The first two rows represent the sequences for the control conditions (Known words, Unlearned words) and the last two rows represent the experimental conditions (Novel words OP, Novel words OPS). Note that “OP” and “OPS” corresponds to set A for half of the participants and set B for the other half. In each condition, words are inserted within pseudowords (PW) every fifth stimulus so that the oddball category (words) appears 2 times in 1 s (at 2 Hz). Sequences lasted 60 s and were presented at 10 Hz.

Inspired by Lochy et al. (2024), the EEG stimulation began with a fixation phase, where participants focused on two vertical blue bars positioned to the left and right of the screen center (−0.3 and 0.3 in unit coordinates) for 1–3 s. To maintain spatially distributed visual attention, participants monitored random color changes of these bars, which remained visible throughout the sequence. The bars changed color for 200 ms, 15 times per sequence, and participants were instructed to press the space key when both bars turned red. This orthogonal task was implemented to enhance word-selective responses by preventing narrow central fixation, as previously demonstrated (Lochy et al., 2024). At the beginning of the sequence, the display of the vertical bars was followed by 2 s stimulation fade-in, followed by 60s of stimulation and 2 s fade-out. The fading-in/out procedure was used to avoid abrupt eye movements or blinks at the beginning or end of a sequence. During the stimulation, an item was presented with a square-wave presentation (Retter et al., 2018) thus, reaching full contrast at once. Every sequence followed a fixed structure (see Figure 3) with items presented at 10 Hz and consisting of base stimuli (B) and periodic oddball stimuli (O) introduced at 2 Hz (every fifth item) such as BBBBOBBBBO (1 s). Base stimuli always consisted of pseudowords, and oddball stimuli consisted of one among 4 conditions: Novel word OP & Novel word OPS (experimental condition), unlearned words, & Known words (baseline conditions). The experimental conditions were created using the Novel words learned with the OP and OPS training methods, respectively. The Known words condition consisted of 16 known words, while the unlearned words condition was created by selecting 16 words from the list of 32 unlearned words (see General Stimuli). Each condition of 16 words was paired with four sets of 16 pseudowords, specifically created for EEG testing (e.g., 64 pseudowords for the 16-word conditions, see Table 1). Stimuli were presented as images using the Verdana font. They were displayed on a screen with a resolution of 1920 × 1,080 pixels. Viewed from 1 m, the average size of the stimuli was approximately 3.97 × 1.26 degrees of visual angle. These stimuli were centered on the screen and were not repeated immediately after being shown. Each condition was repeated four times for a total of sixteen sequences. Every sequence lasted 1 min for a total of 16 min of stimulation. Since we were testing at 10 Hz (10 images/s) with oddball stimuli every 5 items, each sequence contained 600 stimuli among which 120 words and 480 pseudowords, each item being presented with a similar repetition rate. A small break was allowed after each sequence and a longer break after eight sequences (middle of the EEG session). The total duration of the EEG recording session, both at pre-learning and post-learning sessions, was 20 min.

### 3.4 EEG acquisition, preprocessing and analysis

Participants were comfortably seated in a quiet room at a distance of 1 meter from the screen and had their head circumference measured for a selection of an appropriately sized EEG cap. Electroencephalographic activity was acquired at a sampling rate of 1,024 Hz using a 64-channel Biosemi Active II system (Biosemi, Amsterdam, Netherlands) with electrodes including standard 10–20 system locations^3^ as well as an additional row of posterior electrodes (corresponding to standard positions PO9, I1, I2, and PO10). Eye movements were monitored by two additional electrodes placed at the outer canthus and above the right eye. All EEG electrode impedances were held below 30Mv before recording.

EEG data preprocessing was performed in Matlab R2022 (The matworks) using letswave 6/7.^4^ Each subject data file was filtered with a band pass filter between 0.1 and 100 Hz and then segmented into 16 sequences, including 2 s before and after each sequence, resulting in 64 s segments, for visual inspection. Data were also downsampled to 512 Hz. Independent Component Analysis (ICA) was applied to 37.5% of the EEG datasets to correct for ocular artefacts whereas a visual inspection noticed artifact-ridden electrodes. In those cases (2.89%), they were replaced by using linear interpolation with the mean of three neighboring electrodes. EEG recordings were then segmented again into 60-s epochs and re-referenced to the common average. We resulted with one dataset including four repetitions of 60 s per participant and condition.

We followed the standard procedure in the domain (Lochy et al., 2015; Rossion et al., 2020; Volfart et al., 2021). The four trials for each condition were averaged in the time domain (Novel word OP, Novel word OPS, Unlearned words, Known words) reducing the EEG activity that was not phase-locked to the stimulus. Data were then submitted to a Fast Fourier Transform (FFT). After the FFT was performed, the normalized amplitude spectra were extracted for each channel. The absolute value of the FFT was divided by the number of data points to obtain the normalized amplitude spectra. Since the length of the epochs was 60 s, the frequency resolution was quite high (1/60 = 0.0167 Hz) and allowed the identification of the responses to the base stimulation at 10 Hz and to the oddball stimulation at 2 Hz and harmonics (our frequencies of interest). To further reduce potential noise variations across the frequency spectrum, a local baseline-correction was applied (BL). Given that the amplitude at any frequency is a combination of signal and noise (Heinrich et al., 2009), it involved subtracting from each frequency bin the mean of the 20-surrounding bin (e.g., noise, 10 on each side), resulting in a more accurate representation of the data. The signal-to-noise ratio (SNR, see response spectra in the Results section 4.3.) was calculated by dividing the amplitude at each frequency bin by the average amplitude of 20 surrounding bins (10 on each side; Liu-Shuang et al., 2014). Finally, periodic stimulation typically generates EEG responses at the stimulation frequency (e.g., 10 Hz) and harmonics at integer values of the stimulation frequency (i.e., 20 Hz, 30 Hz; etc.) (Norcia et al., 2015; Regan, 1989). It is recommended to consider all significant harmonics in the analysis (Retter et al., 2021; Retter and Rossion, 2016) given that they contribute to the EEG response. Thus, to determine which harmonics were significant both for the base rate response (10 Hz, 20 Hz, etc.), and the oddball response at 2 Hz (2 Hz, 4 Hz, 6 Hz, etc.), *Z*-scores [*Z*(*x*) *= x-mean(noise)/SD (noise)*] were computed per group on the raw (uncorrected for baseline EEG noise) amplitudes, and considered significant if larger than 2.33 (*p* < 0.01, one-tailed, signal > noise).

As concerns the oddball stimulation frequency (2 Hz and harmonics), discrimination responses of words among pseudowords (word-selective responses) were significant (*Z*-score >2.33) in session 2 for novel words OP, novel words OPS and known words (the interested reader can refer to the Results section 4.3. for illustration). For known words, significant responses were observed in both sessions. No discrimination responses were found for unlearned words in any session, and no response occurred in session 1 with novel words (pre-learning), as expected. For the novel words in session 2, the highest number of consecutive significant harmonics was 4 (from 2 Hz to 8 Hz), and 7 for known words in both sessions (excluding the base rate at 10 Hz). Thus, the sum of baseline corrected amplitudes (SBL) was computed on 7 consecutive harmonics (from 2 to 14 Hz, excluding 10 Hz) for each condition to select an identical number of harmonics across conditions. From the ranking of the largest amplitude value of electrodes in all conditions and sessions, five electrodes emerged (P9, PO9, PO7, P7 & I1). This corroborates previous findings in the literature (Lochy et al., 2015) for word-selective responses, therefore we confidently selected the previously defined region of interest (ROI) in the left hemisphere (LH) for further analysis.

## 4 Results

Our analyses are presented into three main sections: performance during the learning procedure, lexical decision tasks, and FPVS-EEG task. First, results from the training tasks provide valuable information regarding the efficacy of the method (OP, OPS) employed throughout the learning procedure. Second, lexical decision tasks provide essential data (reaction time; accuracy) to evaluate changes in processing novel words and enable the assessment of their recognition and their impact on neighbor words (ON1L) and neighbor pseudowords (PW1L). Third, FPVS-EEG tasks track the emergence of neural responses to novel words (pre-learning, post-learning) with neurophysiological data.

### 4.1 Training tasks

A 2 × 4 repeated measures ANOVA with *Method of training* (OPS vs. OP) and *Task* (Visual Matching/Typing /Orthographic Matching/Recognition) was conducted on accuracy and RT.

**Figure 4:**
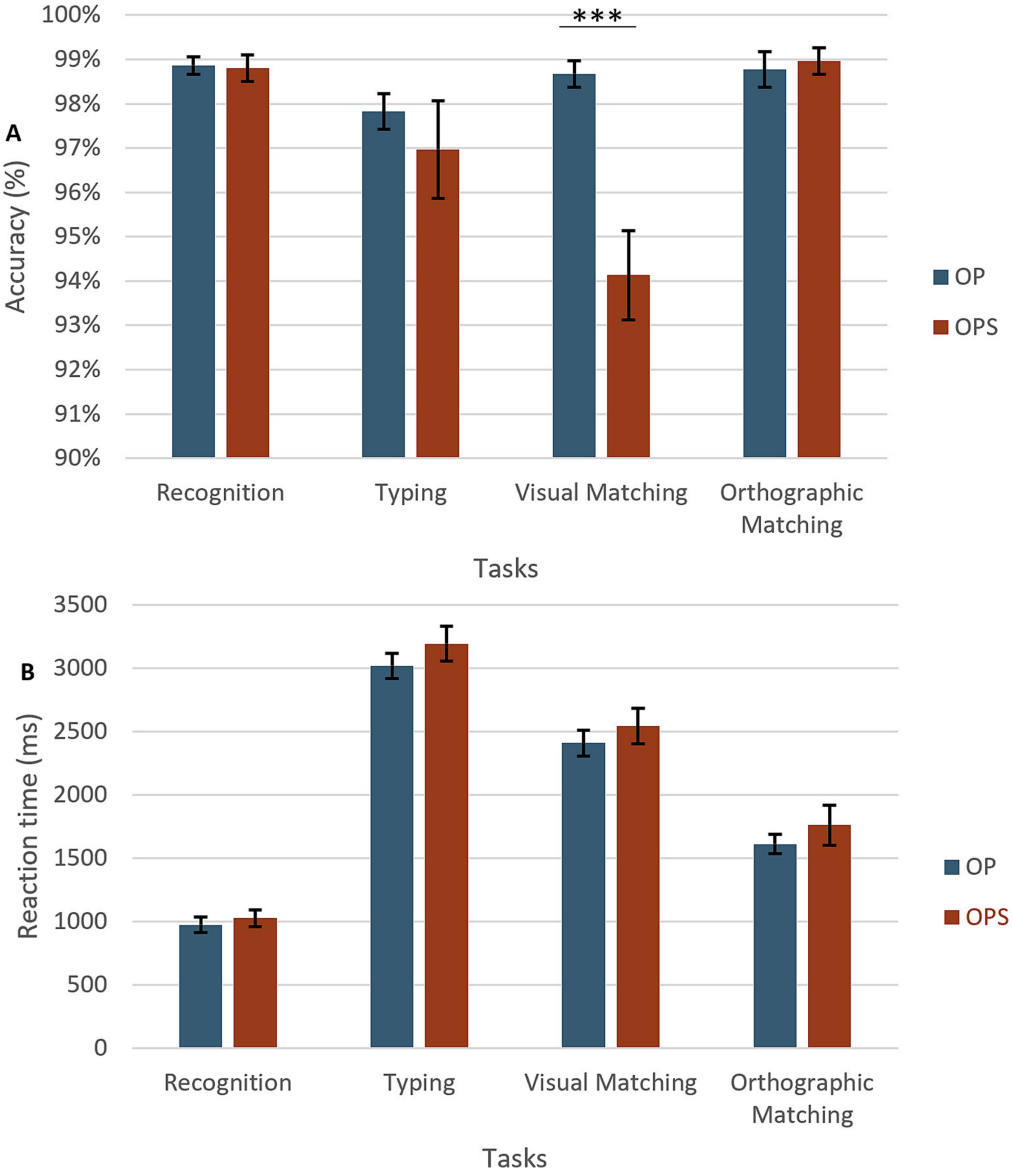
Results of the training tasks. **(A)** Accuracy across the four training tasks (Recognition, Typing, Visual Matching, Orthographic Matching, and Typing) for the two learning conditions. OPS training is shown in red and OP training in blue. **(B)** Reaction times (RTs) for the same tasks and conditions.

For accuracy (see Figure 4A), main effects of *Task* [*F*(3,93) = 7.36, *p* < 0.001] and *Method* [*F*(1,31) = 8.66, *p* < 0.006] were found, with performance being overall better for the OP (*M* = 98.5%) than OPS method (*M* = 97.1%). We also observed a significant interaction between the two factors [*F*(3,96) = 7.97, *p* < 0.001]. The comparison of methods in each task showed that the two methods differed only for the visual matching task [*t*(31) = 4.31, *p* < 0.001] with higher accuracy for OP, where the task was to match target words across upper/lower case (*M* = 98.6%), than OPS method, where the task was to match the target to its corresponding image (*M* = 93.9%).

For reaction times (RT; see Figure 4B), a main effect of *Task* [*F*(3,96) = 210.96, *p* < 0.001] was found. Participants were slower for visual matching (*M* = 3,111 ms) than typing (*M* = 2,479 ms), than orthographic matching (*M* = 1,691 ms), and the recognition task (*M* = 1,008 ms). A main effect of *Method* [*F*(1,31) = 4.57, *p* = 0.04] was also found suggesting that training with the semantic method gave rise to overall slower RT (*M* = 2,136 ms) than training with OP method (*M* = 2008 ms). No interaction was found (*F* > 1).

Concerning the free recall task, no differences were observed in terms of accuracy [*t*(31) = −0.970, *p* < 0.339] or time [*t*(31) = −0.267, *p* < 0.791] between the two methods (OP, OPS).

### 4.2 Learning effects in lexical decision task

Analysis of the lexical decision task provides valuable insights to track the evolution of novel word recognition at pre- and post-tests, and to investigate an advantage of one of the employed methods. It is also crucial to evaluate the effects of having learnt novel words on neighbors. Here we first examine the recognition of the novel words learnt with the two methods (OP, OPS). Then we track their impact on word orthographic neighbors (ON1L) and pseudowords neighbors (PW1L).

Preliminary analyses evaluated performance on baseline control items (unknown pseudowords, filler words, and filler pseudowords) and are provided in the Supplementary material 1.1 and 1.2. These analyses confirm that there are no simple test–retest effects. Indeed, no significant changes were found for known words or filler items between pre- and post-learning sessions, supporting the idea that the effects reported below on novel words and their neighbors reflect genuine learning and lexical integration.

Accuracy and reaction times were analyzed separately. RT exceeding the mean plus three times the standard deviation of the category per condition and session were excluded from the analysis.

Accuracy was analyzed using generalized linear mixed-effects models (GLMM) for binary outcomes, with a logit link function and binomial family, implemented in the lme4 R package. After inspecting the distribution of RTs, a natural logarithmic transformation was applied to normalize the data. Transformed RTs were then analyzed using linear mixed-effects models (LMM), also implemented in lme4.

For clarity, reaction time values are reported in milliseconds (ms) in the text and figures. All models (GLMM for accuracy, LMM for RT) included *Session* (Pre-learning, Post-learning) and *Method* (OP, OPS) as fixed effects, with participants as a random effect. To test the significance of fixed effects, Type III Wald chi-square tests were conducted for each mixed model using the car package.

#### 4.2.1 Assessing learning effects via performance on novel words

At pre-test, 7.2% of novel words were categorized as words (were already known or mistakenly identified). After learning, 90.8% of them were recognized as words, without difference between teaching methods [*t*(31) = 1.07 *p* = 0.30].

The mixed-effects model revealed no significant effect of learning method [*χ*^2^(1) = 0.003, *p* = 0.956] on reaction times for novel words at Session 2, suggesting that words learned through OP (*M* = 826.48; SD = 369.21) and OPS (*M* = 826.39; SD = 404.06) methods were recognized with comparable speed.

#### 4.2.2 Assessing learning via competition effects on neighbors

To recall, we expected an impact of novel words learning on orthographic neighbors (ON1L) and on pseudoword neighbors (PW1L), only if novel words have been stored as novel integrated lexical entries. We also expected stronger effects of words associated with semantics (OPS) than without semantics (OP).

##### 4.2.2.1 Word orthographic neighbors (ON1L)

**Figure 5:**
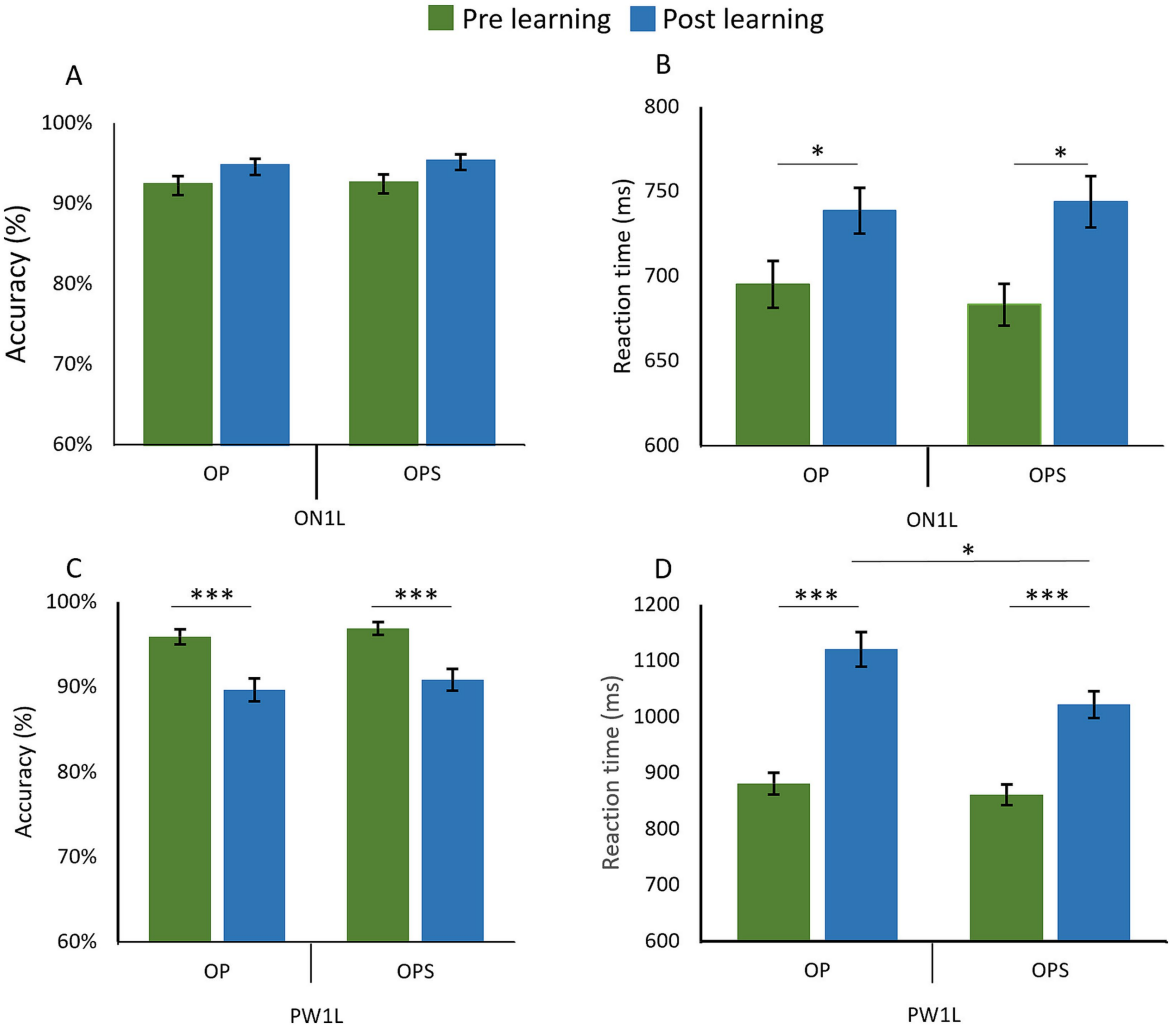
Competing effects on pre-existing orthographic words and pseudoword neighbors in a lexical decision task. Graphs show the change between pre-learning (green) and post-learning (blue) sessions for orthographic neighbors (ON1L) and one-letter-change pseudowords (PW1L) corresponding to items learned with the OP (Orthographic–Phonological) or OPS (Orthographic–Phonological–Semantic) methods. In the top row, Panel **A** displays accuracy (%) and Panel **B** displays reaction times (ms) for orthographic neighbor (ON1L)s. In the bottom row, Panel **C** shows accuracy (%) and Panel **D** shows reaction times (ms) for one-letter-change pseudowords (PW1L).

On accuracy, the GLMM revealed no significant main effect of Session [*χ*^2^(1) = 2.32, *p* = 0.128], no effect of Method [*χ*^2^(1) = 0.18, *p* = 0.668], and no Session × Method interaction [*χ*^2^(1) = 0.06, *p* = 0.80] (see Figure 5A).

For RT (Figure 5B), the LMM revealed a significant main effect of Session [*χ*^2^(1) = 11.31, *p* < 0.001]. Reaction times for orthographic neighbors were significantly slower in post-learning (*M = 751.46 ms; SD = 324.92*) compared to pre-learning (*M = 702.99 ms; SD =* 295.83) (Figure 5B). However, no significant main effect of Method [*χ*^2^(1) = 0.04, *p* = 0.838] or Session × Method interaction [*χ*^2^(1) = 0.02, *p* = 0.900] was observed.

##### 4.2.2.2 Pseudoword neighbors (PW1L)

On Accuracy, the GLMM revealed a significant main effect of Session [*χ*^2^(1) = 14.91, *p* < 0.001] with accuracy being significantly lower in post learning (*M = 0.90; SD = 0.3*) compared to pre-learning (*M = 0.96; SD =* 0.19). However no significant main effect of Method [*χ*^2^(1) = 0.43, *p* = 0.513] and no significant Session × Method interaction [*χ*^2^(1) = 0.14, *p* = 0.708] were observed (Figure 5C).

On RT, the LMM revealed a significant main effect of Session [*χ*^2^(1) = 96.55, *p* < 0.001] with RT being significantly slower in post-learning (*M = 1070.7 ms; SD =* 595.22) compared to pre-learning (*M = 870.85; SD = 419.43*). A significant main effect of Method [*χ*^2^(1) = 11.64, *p* < 0.001] was observed, with RT being significantly slower for the OP method (*M = 996.87 ms, SD* = 572.72) compared to the OPS method (*M* = 939.07 ms, *SD* = 464.53). Additionally, the Session × Method interaction was significant [*χ*^2^(1) = 3.96, *p* = 0.046]. A *post hoc* analysis was conducted to compare the effect of Method at each session. In pre-learning, no significant difference in RT between OP (*M = 880.94 ms; SD = 435.26*) and OPS (*M = 860.82 ms; SD = 403.27*) was found (*p* = 0.51). However, in post-learning, the analysis highlights significantly [*t*(31) = 3.38, *p* = 0.002] slower RT for PW1L matched on novel word OP (*M = 1120.27 ms; SD* = 668.32) compared to the OPS method (*M = 1021.78 ms; SD = 509.02*) (Figure 5D).

### 4.3 FPVS-based EEG frequency analysis: tracking learning effects

Neural responses occur in the response spectra both at the base rate (general visual stimulation frequency, 10 Hz, see part A of each panel in Figure 4) and at the oddball stimulation frequency (2 Hz) and harmonics. A preliminary analysis of the base rate confirms that all conditions elicited similar general visual responses and participants paid equal attention, given that no difference emerged between conditions or sessions (see Supplementary material 2).

The analysis of baseline conditions (known words and unlearned words, Figures 6.1, 6.2) are presented in Supplementary material section 2. As is evident from Figure 6.1, word-selective responses for known words display the classical and expected left-lateralized topography similar to previous studies (Lochy et al., 2015, 2025; Marchive et al., 2025). The strength of response, visible in the SNR response spectrum (Figure 6.1.A) or summed-amplitude spectrum (Figure 6.1.B) does not change between pre- and post-test (details in Supplementary material 2). Concerning Unlearned words, no discrimination response is measured, neither at pre-test nor at post-test (Figure 6.2, Supplementary material 2). These preliminary observations ensure that the test–retest procedure does not, by itself, modify oddball responses in the second session.

**Figure 6:**
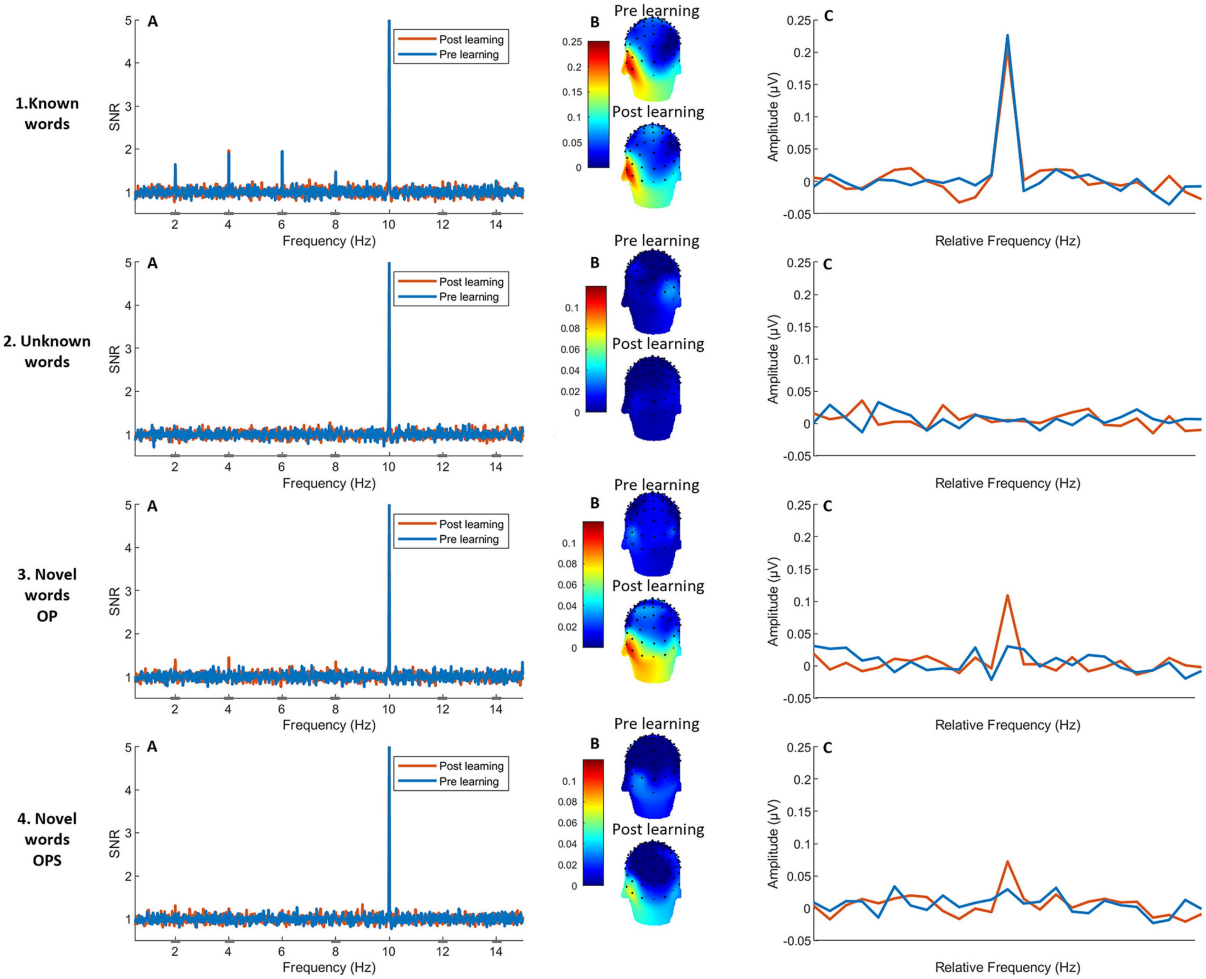
Response spectra and topographies for word-selective responses in the left occipito-temporal ROI. The two upper graphs represent the two control conditions (1: known words and 2: unknown words) and the two inferior graphs represent the two experimental conditions (3: novel words OP and 4: novel words OPS). Each row presents: **(A)** the response spectra with SNR values (1 = chance-level) for word selective responses of electrodes over the left ROI (over the occipito-temporal cortex) at the oddball frequency (2 Hz) and harmonics (4, 6,8,12 and 14 Hz) at pre (blue line) and post-learning (orange line). The high peak at 10 Hz represents the base rate (general visual stimulation) response; **(B)** topographies of the word-selective responses (2 Hz) for the sum of significant harmonics, recorded at pre and post-learning. **(C)** Significant word discrimination responses are displayed in amplitudes (μV) after summing the significant harmonics, centered at the frequency of word stimulation.

To test learning effects in the two experimental conditions (Figure 6, bottom rows), and to assess the impact of learning method, a linear mixed-effects model was conducted with *Session* (pre-learning, post-learning), *Method* (Novel words OP, Novel words OPS), and their interaction.

The linear mixed-effects model for amplitude revealed a significant main effect of *Session* [*χ*^2^(1) = 16.53, *p* < 0.001] with higher amplitudes observed in session 2 (*M* = 0.97 μV, SD = 0.14) compared to session 1 (*M* = 0.56 μV, SD = 0.07). A significant main effect of *Method* was found [*χ*^2^(1) = 7.07, *p* = 0.008], with a higher average amplitude for the OP method (*M* = 0.086, *SD* = 0.092) compared to the OPS method (*M* = 0.067, *SD* = 0.067). The *Session × Method* interaction was at the threshold for statistical significance [*χ*^2^(1) = 3.82, *p* = 0.05]. *Post hoc* comparisons (using estimated marginal means) revealed that in the *post-learning* session, the OP method elicited significantly higher word-selective amplitudes (*M* = 0.1176, SE = 0.0137) than the OPS method (*M* = 0.0767, SE = 0.0137), *t*(93) = 2.66, *p* = 0.009 (for analysis of the distribution of harmonics see Supplementary material 3). In contrast, no significant difference between methods was found in the *pre-learning* session (OP: *M* = 0.0550, SE = 0.0137; OPS: *M* = 0.0567, SE = 0.0137), *t*(93) = −0.10, *p* = 0.92.

## 5 Discussion

The first aim of this study was to investigate immediate lexicalization of novel words with behavioral and neurophysiological measures. To track behaviorally a potential lexical integration, we used a lexical decision task, while the emergence of neural representations for novel words was assessed through an original EEG-FPVS approach. Both behavioral and neurophysiological results provide converging evidence of learning effects, suggesting successful immediate new representation of novel words. Our second aim was to compare two training methods to evaluate the role of adding semantic information to orthography and phonology in novel visual word learning. In one condition, novel words were learned with orthographic and phonologic information only (OP), while in the other, semantic information was added (OPS). The comparison of these methods revealed mitigated findings discussed here below.

### 5.1 Novel words are rapidly learned

#### 5.1.1 Tracking successful learning: novel words in the lexical decision task

Our first key finding concerns the successful creation of lexical representations of novel words, as revealed by both behavioral and neurophysiological evidence. In the lexical decision task, novel words were recognized more accurately after learning (7–90%), in line with previous studies (Bermúdez-Margaretto et al., 2015, 2019; Borovsky et al., 2010, 2012; Lindsay et al., 2012; Partanen et al., 2018). However, due to the short time interval between learning and testing, this recognition may partly reflect episodic memory traces rather than full lexicalization (Batterink and Neville, 2011; Bermúdez-Margaretto et al., 2019; Qiao and Forster, 2013).

Two additional behavioral effects suggest lexical engagement rather than simple episodic traces. First, reaction times to orthographic neighbors (ON1L) were significantly slower after learning, while this effect was not observed for baseline (control) words (Supplementary material 1.1). This could reveal that novel words interacted with the recognition of preexisting neighbors, hence indicating that they were engaged in lexical competition (Bowers et al., 2005; Gaskell and Dumay, 2003; Leach and Samuel, 2007; Qiao and Forster, 2013) and had to be inhibited (McClelland and Rumelhart, 1981).

Second, we observed a strong interference effect on pseudowords neighbors (PW1L): after learning, participants were slower and less accurate in rejecting these pseudowords, compared to pre-learning. This effect was specific, as responses to unrelated pseudowords (fillers) did not change across sessions (see Supplementary material 1.2). These results likely reflect a “word-likeness” effect (Ratcliff et al., 2004): pseudoword neighbors have become word-like because of the novel lexical representations just acquired. Indeed, Morton’s (1969) model posits that pseudowords closely resembling real words are difficult to reject in lexical decision task because they activate similar lexical representations. It is also consistent with the leaky accumulator model (Dufau et al., 2012), whereby strengthened representations of novel words increase their similarity to pseudoword neighbors, making them harder to reject and leading to longer decision and more errors. In both cases, the worse performance for pseudoword neighbors at post-test is directly related to the newly created representations for novel words.

We note that the slower RTs both on orthographic neighbors and PW1L could also be related to the mixed lists that we used in the task, given the well-known list context effects in lexical decision (eg., Lupker and Pexman, 2010). For instance, a context presenting pseudowords that differ from words by only one letter, or contain a transposed-letters pair, may slow down response times to related words (Perea and Lupker, 2004). Thus, such context effects could also play a role here given that both novel words and PW1L differed by one letter from base words. However, the list itself was the same at pretest and post-test. Thus, what we crucially highlight here is a *change* between the two testing sessions, that necessarily relates to the material that has been learnt. Various tasks have been used in the past to assess lexical engagement and competition effects on neighbors (semantic categorization, primed lexical decision, stroop task, pause detection, phoneme/ letter monitoring, etc.; see Palma and Titone, 2021, for a discussion on the variety of tasks), but to our knowledge, no study empirically tested the impact of using a mixed list (thus, including novel words and pseudoword neighbors in the task) rather than a list focusing on base words. Therefore, the competition effects (on orthographic neighbors) and interference effects (on pseudoword neighbors) that we measure in the current experiment still offer support to the idea that novel words could be lexically integrated, beyond simple episodic memory traces.

#### 5.1.2 Neural signatures of learning: FPVS-EEG approach unveils novel word representations

The FPVS-EEG task provided converging neural evidence for the creation of novel word representations. Specifically, FPVS-EEG results revealed implicit and automatic discrimination of novel words among normal pseudowords after learning, a phenomenon not observed before their acquisition. Indeed, before learning, novel words were processed as pseudowords and not discriminated from them. After learning, novel words elicited clear word-selective neural responses, observable at the exact frequency of word presentation (2 Hz and harmonics), and with a left occipito-temporal topography as in previous studies with words (Lochy et al., 2015). Despite the limited spatial resolution of EEG, the scalp topography appears compatible with the involvement of the left occipito-temporal cortex (VOTC) known as a key region for visual word recognition (Cohen et al., 2002; Lochy et al., 2018; Murphy et al., 2019) and word learning (Li et al., 2021; Moore et al., 2014; Taylor et al., 2014). Indeed, several studies have described the VWFA as region rapidly adapting to support both the recognition of familiar words and the acquisition of new word forms (Glezer et al., 2015; Riesenhuber and Glezer, 2017; Tao et al., 2024). Importantly, it has been shown to encode novel orthographic representations even in the absence of semantic information (Glezer et al., 2015; Riesenhuber and Glezer, 2017), highlighting its role in the early stages of the acquisition of new word forms. Consistent with this view, the scalp topographies observed in our study over the left occipito-temporal cortex support the idea that newly learned words engage this region despite the lack of explicit semantic content.

The emergence of word-selective responses post-learning supports the idea that novel words rapidly formed lexical representations in the orthographic lexicon (Lochy et al., 2015, 2024) (although not reaching the level of known words, see Supplementary material 4) given that post-learning increases in neural signal strength within this region indicate the formation of new lexical representations (Bakker-Marshall et al., 2018; Li et al., 2021). Furthermore, these effects cannot be explained by attentional differences between sessions: first, no difference emerged on the two control conditions (known words and unlearned words, Figure 6), and second, base rate responses at 10 Hz (reflecting general visual and attentional processing) remained stable across conditions and sessions (see Supplementary material 2). Our results thus show that FPVS oddball responses are sensitive enough to reflect genuine learning-induced neural changes.

#### 5.1.3 Fast or slow? Investigating immediate learning

Our findings provide new evidence to the ongoing debate on whether lexical engagement requires consolidation. Many studies suggest that lexical competition effects only emerge after a consolidation period (Bakker et al., 2015; Davis and Gaskell, 2009; Gaskell and Dumay, 2003; Takashima et al., 2014; Tamura et al., 2017). However, our results support an alternative view: that lexical engagement can occur immediately after learning, without requiring a consolidation period (Fernandes et al., 2009; Kapnoula et al., 2015; Kapnoula and McMurray, 2016; Lindsay and Gaskell, 2013; Szmalec et al., 2012; Viviani, 2019; Weighall et al., 2017). Our study aligns with this immediate creation of new representations, showing converging behavioral and neural evidence within the same testing session. In a lexical decision task, novel words interfered with orthographic neighbors and impacted pseudoword neighbors processing, potentially reflecting lexical competition (although the alternative account, in terms of list-context effects, remains to be tested in the future). Separately, an FPVS-EEG task revealed word-selective neural responses to novel words, confirming the rapid emergence of neural representations.

### 5.2 Does learning method matter? Investigating OP and OPS training

#### 5.2.1 A surprising behavioral advantage

We compared two learning methods, one providing orthographic and phonological (OP) training, and the other adding semantic information (OPS). Based on previous research (Seidenberg and McClelland, 1989; Angwin et al., 2014; Pexman et al., 2008; Taylor et al., 2011), we hypothesized that OPS would facilitate novel word learning, as richer representations should strengthen encoding and lexical quality (Perfetti and Hart, 2008). However, our findings did not reveal such an advantage of the semantic method.

Behaviorally, recognition performance for novel words did not differ between OP and OPS: both reached high accuracy levels. Lexical competition effects, reflected in slower RTs for orthographic neighbors (real words neighbors and pseudowords neighbors) post-learning were observed regardless of the learning method, suggesting that both methods led to comparable lexical engagement. The only difference between methods that was found concerns the RT increase at post-test for pseudoword neighbors (PW1L), where it was larger for OP (+256 ms) than for OPS (+153 ms), indicating a stronger interference effect in the OP condition. This effect may stem from a methodological difference in training tasks. During the visual matching task, the OPS training displayed four images of concepts while the OP training displayed four pseudowords to choose from. The greater exposure to such neighboring pseudowords during OP training may have increased the uncertainty about the status of other pseudowords in the lexical decision task, making them harder to reject. This could explain the stronger interference effect observed in RTs for OP.

#### 5.2.2 Neural evidence: a trend in favor of OP

Many ERP-EEG studies show that meaningless words or pseudowords elicit stronger N400 amplitudes than known words (Bentin, 1987), as the N400 is a well-established marker of lexical-semantic access (Kutas and Federmeier, 2011). Training with semantic information typically reduces N400 amplitudes (Angwin et al., 2014; Batterink and Neville, 2011; Borovsky et al., 2010, 2012; Mestres-Missé et al., 2007; Perfetti et al., 2005), suggesting that semantic enrichment facilitates new lexical representations and strengthens word learning (Bermúdez-Margaretto et al., 2018, 2019; Frishkoff et al., 2010). We thus expected greater neural word-selective responses for novel words trained with OPS. Contrary to expectations, FPVS-EEG results revealed the opposite, with larger word-selective responses in the OP condition compared to OPS at post-test and not at pre-test. Although the interaction was at the threshold for statistical significance (*p* = 0.050), it deserves to be discussed. Three possible explanations may account for this unexpected finding.

First, the systematic presence of images during OPS training, concomitant to written forms, may have diverted attention from orthographic encoding, weakening word-form representations. In favor of this interpretation, we found that the overall RT during the learning tasks was longer for the OPS items than the OP items. In contrast, OP training displayed only orthographic and phonological forms, potentially leading to stronger neural responses when viewing the orthographic form during testing. This aligns with evidence that simultaneous word-image presentation can divide attentional resources, impairing orthographic learning efficiency (Landi et al., 2006). If this was the case, it suggests that learning a new orthographic form alongside a picture which is common in vocabulary learning may not be optimal for visual word encoding. The simultaneous presence of images may have hindered full orthographic encoding or made it less effective than in the OP condition. Consequently, the word-selective response measured in FPVS-EEG tended to be stronger in OP because during training, attention was directed solely to the written word form.

Second, semantic learning typically unfolds over a longer timeframe than word form learning, reflecting differences in the type of information acquired and the neural mechanisms engaged (Dumay and Gaskell, 2007; Takashima et al., 2014). According to the Complementary Learning Systems (CLS) account (Davis and Gaskell, 2009; McClelland et al., 1995), newly acquired words first rely on hippocampal-dependent episodic traces before gradually becoming integrated into neocortical memory networks, a process that often depends on sleep-based consolidation. Initial learning is therefore more closely tied to the encoding of word forms (Bermúdez-Margaretto et al., 2018, 2019; Gaskell and Dumay, 2003; Lindsay and Gaskell, 2013;). In ERP studies, this stage has been associated with the Late Positive Component (LPC), which reflects episodic memory processes and recognition of previously presented stimuli, and which increases even after simple visual repetition of pseudowords (Bermúdez-Margaretto et al., 2018, 2020). Importantly, several studies indicate that semantic benefits in word learning may only emerge after consolidation (Angwin et al., 2014; Bakker et al., 2015; Takashima et al., 2017), consistent with the CLS framework. Indeed, semantic enrichment effects are often observed after a delay, particularly following sleep (Kurdziel and Spencer, 2015; Schimke et al., 2021; Takashima et al., 2014), or at least change over time (Smejkalova and Chetail, 2024). This view contrasts with evidence of immediate semantic benefits (Bermúdez-Margaretto et al., 2018, 2019; García-Gámez and Macizo, 2022), suggesting that the presence or absence of such effects may depend on the timeframe of assessment. Given that participants in our study were tested immediately after learning, the delayed advantage of OPS training may not yet have had the opportunity to manifest.

Third, semantic processing may engage neural regions not optimally captured by the FPVS-EEG approach used here. While lexical and orthographic processing are typically associated with occipito-temporal regions, semantic processing is often linked to more anterior brain areas, such as the anterior temporal lobe (Ralph et al., 2016) and frontal regions (Frishkoff et al., 2010; Volfart et al., 2021). If OPS preferentially engaged these anterior regions, this could explain why no increased neural response was observed in the occipito-temporal region targeted by FPVS-EEG. However, known words, which also carry semantic content, do not elicit word-selective responses with a different topography, neither here (Figure 6) or in previous studies (Lochy et al., 2015), suggesting that semantic content does not necessarily alter the neural response localization in this paradigm. It is worth noting that previous FPVS studies investigating semantic categorization have used slower stimulation frequencies than those employed in the present study. For example, Stothart et al. (2017) demonstrated robust oddball responses to semantic categories using a 6.25 Hz paradigm, with distinct topographical patterns observed across image sets. Animals versus non-animals elicited a left-lateralized centro-parietal cluster, while birds versus non-birds showed both central and parieto-occipital activity. Importantly, these effects remained significant after controlling low-level visual features, confirming their semantic origin. Similarly, Volfart et al. (2021) revealed automatic, frequency-tagged neural responses at 1 Hz (with a 4 Hz base frequency) when categorizing written words contrasting living and non-living entities, indicating that conceptual categorization occurs implicitly at a slow frequency. In the same vein, alternating text vs. scrambled text at different frequencies revealed that the response was strongest at 1 Hz and it was the most left lateralized at 4 Hz (Yeatman and Norcia, 2016). Furthermore, the brain could not track the difference between text/scrambled images anymore at 9 Hz, although text vs. single word processing (as we do here) presumably involves higher-order and more complex cognitive processes. Other studies have identified 4 Hz as an optimal base frequency for eliciting oddball lexical responses (Marchive et al., 2025), a rate that aligns with the average silent reading speed in adults (Brysbaert, 2019). Taken together, these findings suggest that semantic effects may emerge more robustly at slower stimulation rates than those used in the present paradigm. Future research should explore whether adjusting the presentation frequency could enhance sensitivity to semantic processing and better reveal method-related differences in word recognition.

Together, these arguments suggest that the absence of a stronger neural response for OPS does not necessarily indicate that semantic training was ineffective. Instead, differences in attention allocation, consolidation dynamics, or neural measurement sensitivity may have influenced the observed results. Further research is needed to determine whether semantic effects emerge after consolidation or require alternative neurophysiological approaches for detection.

To conclude, our findings highlight for the first time that the FPVS-EEG approach is sensitive to immediate learning changes and the formation of novel representations. Behaviorally, results suggest that novel words interacted with preexisting lexical knowledge and impacted decisions both on orthographic and pseudoword neighbors. This suggests that pseudoword neighbors could also serve as a valuable tool for further investigating lexicalization. Comparing the two training methods, we found no advantage of adding semantics but we provide interpretation of this result possibly due to divided attention when images were presented alongside words, that lead to weaker word-form encoding.

Finally, this study raises key questions for future research. First, examining the persistence of novel word recognition over time would provide stronger evidence of lexicalization. Second, further investigation is needed to determine when and how semantics enhances learning, with FPVS-EEG offering a promising, sensitive, and implicit approach to address this question.

## Data Availability

The datasets presented in this study can be found in online repositories. The names of the repository/repositories and accession number(s) can be found at: https://osf.io/syn7t/?view_only=9e282d08d97849549e0be48285b722ab.

## Footnotes

^1^ https://pstnet.com

^2^ https://acapela-box.com

^3^ http://www.biosemi.com

^4^ https://letswave.cn/
