# Supplementary material for: Tracking novel visual word learning via different methods with an original FPVS-EEG approach
Source: Front Hum Neurosci. 2025 Oct 20;19:1647925. doi: 10.3389/fnhum.2025.1647925 (PMC12580203; doi:10.3389/fnhum.2025.1647925)
Supplement: Supplementary file 1 [file Data_Sheet_1.docx]

**Tracking novel visual word learning via different methods with an original FPVS-EEG approach**

**SUPPLEMENTARY MATERIALS**

#### **Control Analyses**

A series of complementary analyses were performed to assess whether the changes highlighted between pre- and post-test could have been due to the test-retest nature of our design.

#### **Lexical decision task**

To recall, our analyses reveal slower reaction times on orthographic neighbors of novel words (ON1L), and on pseudoword neighbors (PW1L) after learning. We interpret this as a consequence of learning novel words and lexical integration and investigate here whether this RT increase does not occur on filler items.

We compared in the following analysis experimental items with baseline items (PW1L vs. pseudoword fillers; ON1L vs. word fillers). This analysis could not include the factor Method, as the baseline items cannot be compared across methods (they have not been built to match novel words in each method).

Separate analyses were carried out to examine changes in accuracy and reaction times. Accuracy was analyzed using a generalized linear mixed model (GLMM) with a binomial distribution and a logit link function, while reaction times were examined using a linear mixed model (LMM) applied to log-transformed reaction times. Subjects were included as random factors, and fixed effects were calculated for *Session* and *Type* (experimental vs baseline).

*1.1 ON1L vs word fillers.*

As concerns the accuracy, the GLMM model showed no significant main effects of *Session* (p = .24) , *Type* (p = .51) nor a significant interaction between *Session* and *Type* (p = .31).

On reaction times, the analysis revealed no significant main effect of *Session* (p = .45). However, a significant main effect of *Type* was observed (Estimate = -0.0369, SE = 0.0115, t = -3.22, p < 0.01), with ON1L *(M = 711.39ms; SD = 292.59*) items having slower reaction times than word fillers (*M = 701.95ms; SD = 257.36*). Furthermore, a significant interaction between *Session* and *Type* was found (Estimate = 0.0712, SE = 0.0161, t = 4.41, p < 0.001). A post hoc pairwise comparison was conducted to analyze the *Type* separately and revealed that no effect of *Session* was found (p= .56) for word fillers. However, for ON1L, *Session* effect was significant (t(31) = -2.83 p = 0.008) as described in the article.

*1.2 PW1L vs Pseudowords fillers.*

Concerning accuracy, the GLMM model revealed no significant main effects of the factor *Session* (p = .58) or *Type* (p = .73). However, a significant interaction between *Session* and *Type* was observed (Estimate = - 1.0079, SE = 0.2924, z = -3.45, p < 0.001). A post hoc analysis was conducted to examine changes in accuracy, resulting in pairwise comparisons assessing differences between *Types*.

For pseudoword fillers, no effect of *Session* was found (p = .53) whereas a significant difference (t(31)= 5.64, p < 0.001) was found for PW1L with worse performance in post-learning (M = 0.90ms; SD = 0.3) compared to pre-learning (M = 0.96ms; SD = 0.18).

For RT, the LMM highlighted a significant main effect of *Session* (Estimate = -0.0629, SE = 0.0135, t = -4.68, p < 0.001), with reaction times being faster in pre-learning (*M = 924.37ms; SD = 627.1*) compared to post-learning (*M = 944.62ms; SD = 520.43*). A significant main effect of *Type* (Estimate = -0.0317, SE = 0.0144, t = -2.21, p = 0.027) was also observed, with PW1L (*M = 967.83ms; SD = 521.84*) eliciting slower reaction times than pseudowords fillers (*M = 908.88ms; SD = 614.7*). Additionally, a significant interaction between *Session* and *Type* was found (Estimate = 0.2448, SE = 0.0205, t = 11.94, p < 0.001). Indeed, no significant *Session* effect (p = .79) was found for pseudoword fillers whereas for PW1L, RTs significantly increased (t(31)= -5.494, p < 0.001) from pre-learning (*M = 870.85ms; SD = 419.42*) to post-learning (*M = 1070.7ms; SD = 595.22*) as highlighted in the previous analysis.

Thus, both for words and for pseudowords, this complementary analysis confirms that the observed effects are specific to neighbors of the novel words just acquired, and are not due to test-retest.

#### **EEG data**

To assess test-retest reliability, separate linear mixed-effects models were conducted for each method (Known words, Unknown words) with Session (pre-learning vs. post-learning) as a fixed effect and Subjects as a random intercept. In the Known words condition, the model revealed no significant effect of Session (p = 0.364), indicating stable amplitude across sessions. A similar pattern was observed in the Unknown words condition, where amplitude did not differ between sessions (p = 0.801). These findings suggest strong test-retest reliability across methods, with no significant changes in amplitude over time

**Base rate responses**

As concerns base rate responses, in all conditions (Novel word OP, Novel word OPS, unknown words and known words) and sessions (session 1 and 2) significant responses (Z score >2.58) were found at exactly 10 Hz and 3 harmonics (20, 30 and 40Hz) as hypothesized. The sum of baseline subtracted amplitudes (SBL) was computed on the highest number of consecutive harmonics. Congruent with past studies (e.g., Lochy et al., 2015), the ranking of the largest amplitude value of electrodes in all conditions and sessions highlighted three electrodes: O1 (M = 0.61 µV), O2 (M = 0.65 µV) and Oz (M = 0.63 µV). Based on the mean of these electrodes, a medial-occipital ROI was defined for further analysis. These analyses confirmed that no differences in base rate responses were observed across conditions or sessions. This supports the interpretation that the word-selective responses reflect learning effects rather than general variations in visual processing or attention.

A linear mixed-effects model was conducted to examine the effects of Session (pre-learning vs. post-learning) and Method (known words, unknown words, novel words OP, novel words OPS) on the amplitude of the medial occipital ROI. The goal was to determine whether differences in word-selective oddball responses could be attributed to general factors such as attention variation.

The analysis revealed no significant effect of Session (p = 0.463), Method (p > 0.05 for all comparisons), or their interaction (p > 0.05). These findings indicate that amplitude remained stable across sessions and methods, suggesting comparable attention levels and general visual processing across conditions.

1. **Analysis of the distribution of responses across harmonics**

The “known words” condition generates up to 7 significant harmonics, while the novel words conditions generate only 4 significant harmonics in session 2. Although from the frequency domain we can only guess what happens in the time-domain, this might suggest a more complex electrophysiological signal in the known words condition, given that higher harmonic responses may be accounted for in relation to the complex (i.e., non-sinusoidal) responses of the brain (Retter et al., 2021). Indeed, the simplest response -a perfect sinusoid- would concentrate its power on only one harmonic in the frequency-domain, while complex responses containing sharper edges (rise/decay differing from a sinusoid) distribute over multiple harmonics (Regan, 1989; Zhou, Melloni, Poeppel, & Ding, 2016).Thus, higher harmonics are present when complex brain responses are present.

We have computed the percentage of signal captured by each harmonic for the known words, novel words OP and novel words OPS (we did not look at the unlearned words given that there is no discrimination response), over the 7 harmonics used to compute the sum of baseline corrected amplitudes. It is displayed in Supp Fig. 1

**Supp. Fig1**. Distribution of signal response across the 7 harmonics (in percent of the total response).

Descriptively, the known words condition displays less power at the first harmonic (26%) than the two learning conditions (41% and 48%). The response for known words being more distributed across harmonics suggests a more complex response, with a larger number of higher-frequency components involved (for a comparison of children to adults’ brain responses, see Lochy, Schiltz and Rossion, 2019, and Van de Walle de Ghelcke et al., 2020). It could also reflect that there is a longer response latency for known words. Concerning the two experimental conditions, no obvious difference in the pattern of harmonics stems out, except perhaps that the 6^th^ harmonic (12Hz) contains more noise for the OPS condition (but bearing in mind that only 4 harmonics were significant for these 2 conditions).

1. **Does the response for novel words reach the level of known words?**

To assess whether our experimental conditions reached the amplitude level of known words in post-learning, a linear mixed-effects model was conducted with Method (Novel word OP, Novel words OPS, Known words) as a fixed effect and Subjects as a random intercept.

Known words exhibited significantly higher amplitude than both Novel words OP (p = 0.007) and Novel words OPS (p < 0.001), suggesting that neither of the novel word conditions reached the amplitude level of known words in the post learning session.
